# Pathway-Affecting Single Nucleotide Polymorphisms (SNPs) in *RPS6KA1* and *MBIP* Genes are Associated with Breast Cancer Risk

**DOI:** 10.31557/APJCP.2020.21.7.2163

**Published:** 2020-07

**Authors:** Ghadah Shareefi, Alaa Nabil Turkistani, Ahmed Alsayyah, Haitham Kussaibi, Maha Abdel Had, Khaled R Alkharsah

**Affiliations:** 1 *Department of Microbiology, College of Medicine, Imam Abdulrahman Bin Faisal University (IAU), Dammam, Saudi Arabia. *; 2 *Department of Pathology, College of Medicine, Imam Abdulrahman Bin Faisal University (IAU), Dammam, Saudi Arabia. *; 3 *Breast Division, Department of Surgery, College of Medicine, Imam Abdulrahman Bin Faisal University (IAU), Dammam, Saudi Arabia. *

**Keywords:** Breast cancer, SNP, pathway, MAPK, Saudi Arabia

## Abstract

**Background:**

Genetic mutations and polymorphisms play an important role in the transformation of primary cells to malignant cells as it may lead to disturbance of vital pathways regulating cell cycle, DNA damage repair, and apoptosis. In this study, we genotyped single nucleotide polymorphisms (SNPs) which were predicted to affect certain pathways and to increase the risk of breast cancer.

**Methods:**

The study included 81 Saudi breast cancer patients and 100 matching healthy controls from the Eastern Province in Saudi Arabia. The following *SNPs* (*rs3168891, rs2899849, rs2230394, rs2229714*) were then genotyped by TaqMan genotyping assay and the allele and genotype distribution was compared.

**Results:**

The minor allele frequency of the following *SNPs* (*rs3168891, rs2899849, rs2230394, rs2229714*) was T=0.17, A=0.28, A=0.22, and G=0.16 respectively. The G allele of the SNP rs3168891 was significantly associated with increased breast cancer risk (P = 0.00001) while the T allele of the same locus was associated with reduced risk of breast cancer in both heterozygous and homozygous states. The T allele of *SNP rs2229714* which is located in the RPS6KA1 gene was also significantly associated with the increased risk of breast cancer. However, the* rs2899849 SNP *located in the Integrin beta-1 (*ITGB1*) gene was not associated with the increased risk of breast cancer in our study population. Haplotype analysis revealed the presence of three risk haplotypes that increases the risk of breast cancer (*TGGT, TGTA, GATA*).

**Conclusion:**

We showed that three, previously untested, SNPs are associated with increased risk of breast cancer in our population. This may be added to the list of factors involved in breast cancer risk assessment studies. The benefit and the utility of the in-silico prediction of disease risk factors and their genetic association had been demonstrated in this study, yet the predicted risk alleles have to be tested in clinical studies.

## Introduction

The latest Global Cancer statistics indicates that breast cancer was expected to account for a quarter of cancer cases amongst women in 2018 (Bray et al., 2018). Genetic predisposition plays a crucial role in breast cancer development. Up to 10% of breast cancer cases can be attributed to hereditary factors comprising personal or family history of breast or ovarian cancer and inherited gene mutations such as *BRCA1 *and *BRCA2* among others (Eccles et al., 2013; Bray et al., 2018). Genetic mutations and polymorphisms in the human genome could also be linked to the risk of sporadic breast cancer especially in those genes related to cell development and differentiation. One of the early attempts to investigate the correlation between* p21* and* bcl2 SNPs *with susceptibility to breast cancer showed that *p21* was more commonly expressed in breast cancer patients and* bcl2* polymorphism was likely to be associated with breast cancer risk (Johnson et al., 2008). Interestingly, hundreds of SNPs have been associated with breast cancer risk so far (Turnbull et al., 2010; Eccles et al., 2013). 

Genome-wide association studies (GWAS) facilitated the identification of a large number of breast cancer susceptibility alleles with a much lower size effect (Easton et al., 2007; Stacey et al., 2007; Turnbull et al., 2010; Michailidou et al., 2013). Although GWAS studies have shed light on the discovery and validation of novel disease-causing gene mutations and polymorphisms, it remains a challenge to prove the link between the identified risk allele and the biological mechanism of its involvement in the disease process (Stacey et al., 2007). 

Since the transformation of breast epithelial cells to malignant cells involves the disturbance of vital pathways regulating cell cycle, DNA damage repair and apoptosis, Lee et al. designed software to identify the causal SNP from GWAS and their candidate affected pathway in an attempt to link the causal SNPs to a hypothetical mechanism of the disease (Lee et al., 2014). They identified several candidate SNPs among which four are with very strong putative biological mechanisms located in *RPS6KA1*, *ITGB1*, and *MBIP* genes, which modify the growth hormone signaling, PTEN pathway, and the mitogen-activated protein kinase (MAPK) pathways respectively (Lee et al., 2014).

A previous study from Saudi Arabia showed that breast cancer was the ninth prominent cause of death in women (Memish et al., 2014). In contrast to the global decrease in Breast cancer mortality, the incidence of breast cancer has shown a steady incline in Saudi Arabia, with the Eastern province reporting the highest incidence in the country (El Bcheraoui et al., 2015). Previous studies have found an association of breast cancer with multiple variants and SNPs among the Saudi population. A mutation variant within the 3’-untranslated region (3’-UTR) of the *BRCA*1 gene and the *VEGF -2578AA* genotype were found to be associated with increased risk of breast cancer in among Saudi females (Al Balawi et al., 2018; Mir et al., 2018b). While other allele variants such as the progesterone receptor gene mutations and the microRNA-423 T allele were associated with advanced stage and metastasis of breast cancer (Mir et al., 2018a; Albalawi et al., 2020). 

We sought in this study to identify the correlation between SNPs (*rs3168891*, *rs2899849, rs2230394, rs2229714*) and breast cancer risk among the Saudi population. These SNPs have been previously identified in GWAS in the following genes *RPS6KA1* (*rs2229714*), *ITGB1* (*rs2230394*), and *MBIP* (*rs3168891* and *rs2899849*) (Lee et al., 2014). These SNPs are expected to play a role in pathways that are vital for cell survival and development. 

## Materials and Methods

*Patients and Methods*

*Patients and controls*

Saudi female patients diagnosed with breast cancer or with a history of breast cancer regardless of their treatment regimen were recruited for the study. Patients were attending the breast cancer unit at King Fahd Hospital of the University (KFHU) in Alkhobar for interventional treatment or follow up. The targeted control group was Saudi females over the age of 40 years with no history of breast cancer or familial history of breast cancer up to second-grade relatives.

Informed consent was obtained from all patients and control individuals. The ethical approval for the study was obtained from the Institutional Review Board at Imam Abdulrahman Bin Faisal University (number IRB-UGS- 2017-01-092).

*DNA extraction*

Blood samples were collected in 3ml EDTA vacutainer tubes. DNA was then extracted using DNA extraction kit from Qiagen according to the instructions (Qiagen, Hilden, Germany). The quality and quantity of extracted DNA were measured by NanoDrop 2000 spectrophotometer. DNA was stored at -20^o^C till the time of analysis.

*SNP genotyping*

**Figure 1:**
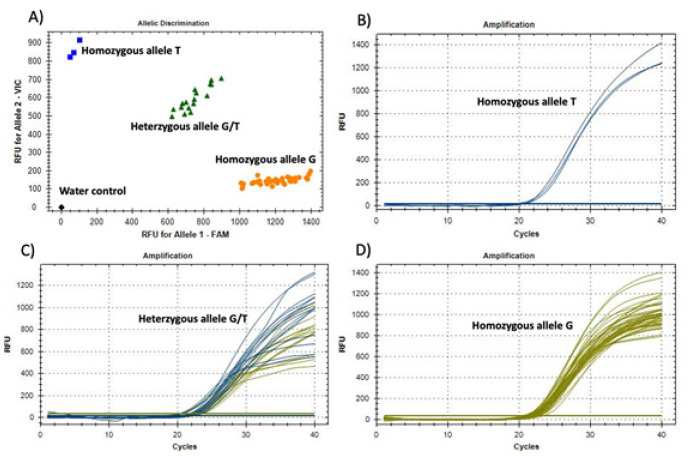
A representative result for the SNP rs3168891 (G/T) genotyping using TaqMan SNP assay. A) Shows the allelic discrimination plot. B) Shows amplification curves for the homozygous allele T. C) Shows amplification curves for the heterozygous allele (G green/T blue). D) Shows amplification curves for the homozygous allele G

Four SNPs (rs3168891, rs2899849, rs2230394, rs2229714) were selected from the publication which described and used the prediction software ICSNPathway to predict the pathway associated SNPs that may affect the risk of breast cancer from genome-wide association studies (Lee et al., 2014). The targeted SNPs were genotyped using TaqMan SNP genotyping Assay (Life Technologies, CA, USA). The real time-polymerase chain reaction (RT-PCR) was performed in 25ul reactions and was run on the thermocycler 7500Fast from applied Biosystems (Thermofisher Scientific, USA) according to the manufacturer’s instruction. Figure 1 shows an example of the SNP genotyping results. The final allelic discrimination of each SNP was performed by the genotyping software associated with thermocycler 7500 version 2.0.6 from ABI (Figure 1). Each allele will be detected with a probe bound to certain fluorescence dye. The presence of the alleles will be reported based on the amplified signal detection of the corresponding dye and then separated the on a plot representing on one axis the first allele and on the other axis the second allele (Figure 1). In case of heterozygous genotype, both alleles will be detected (Figure 1). 

*Statistical analysis*

SPSS software version 24 was used to calculate the Chi-square. Logistic regression was used to estimate the association between SNPs and the risk of breast cancer at a confidence interval of 95%. All studied SNPs were found to be consistent with Hardy-Weinberg equilibrium. Linkage disequilibrium (LD) and the risk of the possible haplotypes were analyzed using the HaploView software version 4.2. A P value of less than 0.05 was considered significant.

## Results

*Study population*

Eighty-one breast cancer female patients and one hundred control female individuals were recruited to the study. The age range for the patients was 26-79 years (median 50) at the time of diagnosis and for the control was 40-78 years (median 51). Patients’ diagnostic parameters are summarized in Table 1. 

*Association between the studied SNPs and Breast cancer*

There was a statistically significant association between SNP rs3168891 and the risk of breast cancer. The G allele at this location was significantly associated with increased risk of breast cancer (OR = 4.1, P = 0.00001) (Table 2) while the T allele of the same locus was associated with reduced risk of breast cancer in both heterozygous and homozygous states (Table 2). Figure 1 shows a representative result for the rs3168891 genotyping with TaqMan Assay. The T allele of the *rs2899849 SNP* was associated with risk of breast cancer only in the homozygous state (Table 2). No association was found between *rs2230394* which is located in the *IBTG1* gene and the risk of breast cancer (Table 2). The T allele of *SNP rs2229714* which is located in the *RPS6KA1* gene was also significantly associated with increased risk of breast cancer (Table 2).

*Association of studied SNPs with patient clinical parameters*

There was no significant statistical association between any of the studied SNPs and other biometric parameters such as age at diagnosis, stage or grade of cancer, or the expression of hormone receptors. 

*Haplotype analysis*

**Figure 2.**
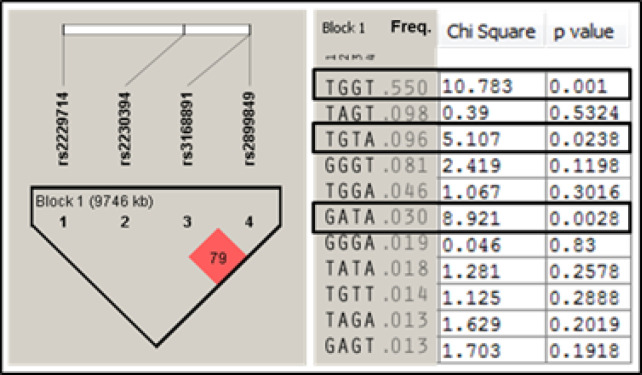
Haplotype Analysis of the Studied SNPs. The left panel shows linkage disequilibrium between SNPs 3 (rs3168891) and 4 (rs2899849) (LOD score > 2), which are located adjacent to each other in the MBIP1 gene but not with any of the other SNPs. The middle panel shows the frequency of the haplotypes in the study population. The right panel shows the statistical association between these haplotypes and breast cancer. The bolded boxed shows the haplotypes significantly associated with breast cancer

Analysis of the haplotypes in the study revealed that three haplotypes are associated with increased breast cancer risk in our study (Figure 2). The haplotypes are TGGT (p-value 0.001), TGTA (p-value 0.238), and GATA (p-value 0.0028) (Figure 2). These genotypes were generated from the studied SNPs in the following order rs2229714, rs2230394, rs3168891, and rs2899849. There was linkage disequilibrium between SNPs rs3168891 and rs2899849 (LOD score > 2), which are located adjacent to each other in the *MBIP1* gene but not with any of the other SNPs.

## Discussion

Breast cancer is emerging as a common female cancer in Saudi Arabia, with a higher incidence reported in the Eastern Province. Multiple studies investigated the association of specific SNPs with breast cancer risk. A significant association was found between breast cancer and the GG genotype of the *rs1799950 SNP* which is located in the *BRCA1* gene (Merdad et al., 2015). The *TP53* gene, a tumor suppressor gene, is one of the most commonly downregulated genes among cancers in humans, including breast cancer (Al-Qasem et al., 2011). A study from Riyad revealed a significant association between R72P located in exon 4 of the *TP53 *gene and the early start of breast cancer among the Saudi population (Al-Qasem et al., 2012). In particular, the RP genotype was found to be protecting against breast cancer (Al-Qasem et al., 2012). However, all studies focus on genes involved in DNA repair machinery, while the transformation of breast epithelial cells to malignant cells may involve the disturbance of vital pathways regulating cell cycle and apoptosis. Lee et al. designed software called “ICSNPathway” to identify the causal SNP from GWAS and their candidate affected pathway in an attempt to link the causal SNPs to a hypothetical mechanism of the disease biology (Lee et al., 2014). They identified several candidate SNPs among which four are with very strong putative biological mechanisms located in *RPS6KA1*, *ITGB1*, and *MBIP* genes, which modify the growth hormone signaling, PTEN pathway, and the mitogen-activated protein kinase (MAPK) pathways respectively (Lee et al., 2014). 

This current study focuses on the correlation of the following *SNPs*; *rs3168891*, *rs2899849*,* rs2230394*, and *rs2229714* predicted by the ICSNPathway software with breast cancer risk in Saudi patients. It is important to note that none of these SNPs were previously described to be associated with the risk of cancer. 

The* SNPs rs3168891* and *rs2899849* are located adjacent to each other in the *MBIP* gene, which affects the MAPK pathway. Both SNPs were found to be associated with breast cancer in our study on both allele and genotype levels. The association was stronger with *rs3168891*. The association with *rs2899849* seems to be influenced by rs3168891 because they are in linkage disequilibrium (LD) (Figure 2). MAPK pathway regulates many vital cellular functions such as cell differentiation, cellular growth, and apoptosis and the disruption of this pathway plays a role in tumorigenesis (Aguirre-Ghiso et al., 2003; Giltnane and Balko, 2014). 

The *SNP rs2229714* was also significantly associated with breast cancer in our study. This SNP is located in the ribosomal protein S6 kinase *A1* gene (*RPS6KA1*), which encodes a serine/threonine kinase. The *RPS6KA1* is one of the S6 kinase family of proteins (Li et al., 2012). The ribosomal S6 kinase family of proteins is the downstream targets of the MAPK pathway effectors and in their turn phosphorylate several regulatory proteins involved in cell growth and differentiation (Casalvieri et al., 2017). Currently, several members of this family are considered promising targets for antiviral therapy (Casalvieri et al., 2017). Furthermore, these proteins are implicated in growth hormone signaling which plays an important role in breast normal physiology through driving the differentiation of ductal epithelium and normal proliferation secretory functions of lobular epithelial cells and disruption of these functions would promote tumor formation (Xu et al., 2011).

The rs2230394 was not associated with breast cancer in our study population. This SNP is located in the gene encoding integrin subunit beta 1 (ITGB1) and was expected to affect the *PTEN* and has04360 pathways. Both pathways are expected to play an important role in breast cancer (DeGraffenried et al., 2004; Luu et al., 2013). However, this SNP seems not to play an important role in the genetic predisposition of breast cancer in our population. Haplotype analysis showed that the most frequent haplotype in the study population indeed consists of the risk alleles of the three SNPs associated with breast cancer in the study (Figure 2).

In conclusion, breast cancer is emerging as an alarming health burden in Saudi Arabia. Extensive studies should be directed towards identifying the factors that predispose to its increased incidence and discovering the pertinent risk factors. We showed that three previously untested SNPs are associated with breast cancer in our population. This may be added to the long list of factors involved in breast cancer risk assessment studies. The benefit of the utility of the software in the prediction of disease risk factors and the genetic association had been demonstrated in this study, yet, on a cautionary note, the predicted risk alleles have to be tested in clinical studies.

**Table 1:** Clinical Data of Breast Cancer Patients in the Study

	N= 81 *	%
Age	77	
less than 40	16	20.8
more than 40	61	79.2
Tumor stage	67	
Early (O, I and II)	51	76.1
Late (III and IV)	16	23.9
Tumor grade	67	
Grade I	8	11.9
Grade II	30	44.8
Grade III	29	43.3
Estrogen receptor expression (ER)	70	
positive	48	68.6
negative	22	31.4
Progesterone Receptor expression	70	
positive	38	54.3
negative	32	45.7
Her2/neu proteins expression	68	
positive	30	44.1
negative	38	55.9
Distant Metastasis	69	
positive	4	5.8
negative	65	94.2

*Data from some patients is missing in each category

**Table 2:** Allele and Genotype Analysis of the Studied SNPs Revealed an Association between the Pathway Affecting SNPs in MBIP and *RPS6KA1* and Risk of Breast Cancer

	Allele/Genotype	Patients N=81	Controls N=100	*P*-value	OR (95% CI)
MAP3K12 binding inhibitory protein (*MBIP*)					
rs3168891	G	150	150	0.00001	4.1 (2.2-8.4)
	T	12	50		
	GG	69	54	0.00001	4.9 (2.4-10.4)
	TG	12	42	0.0001	0.2 (0.1-0.5)
	TT	0	4	0.04*	undefined
rs2899849	T	133	145	0.042	1.7 (1.1-2.9)
	A	29	55		
	TT	55	52	0.03	2.0 (1.1-3.6)
	TA	23	41	0.1	0.6 (0.3-1.1)
	AA	3	7	0.36	2.0 (0.5-9.6)
Integrin subunit beta 1 (*ITGB1*)					
rs2230394	G	137	157	0.18	1.5 (0.9-2.6)
	A	25	43		
	GG	56	60	0.26	1.5 (0.8-2.8)
	GA	25	37	0.47	0.8 (0.4-1.4)
	AA	0	3	0.16	undefined
Ribosomal protein S6 kinase A1 (*RPS6KA1*)					
rs2229714	T	147	158	0.002	2.6 (1.4-5.0)
	G	15	42		
	TT	67	62	0.002	3.0 (1.5-6.1)
	TG	13	34	0.01	0.4 (0.2-0.8)
	GG	1	4	0.3	0.3 (0.01-2.5)

*Mid-P exact test

## Data Availability

All data are available from the corresponding author upon request.

## Declarations

Funding: This project was funded by Deanship for Scientific Research at Imam Abdulrahman Bin Faisal University [Project number 2016-575-Med]. The role of the funding organization was purely financial and did not interfere with the study or the publication process.
